# The power of proximity: Effects of a multidisciplinary fibroid clinic on inter-specialty perceptions and practice patterns

**DOI:** 10.1371/journal.pone.0263058

**Published:** 2022-01-25

**Authors:** Eric J. Keller, Kayla Nixon, Lola Oladini, Howard B. Chrisman, Angela Chaudhari, Magdy P. Milad, Robert L. Vogelzang

**Affiliations:** 1 Division of Interventional Radiology, Stanford University, Stanford, CA, United States of America; 2 Division of Minimally Invasive Gynecologic Surgery, Northwestern University, Chicago, IL, United States of America; 3 Division of Interventional Radiology, Northwestern University, Chicago, IL, United States of America; Waikato Institute of Technology, NEW ZEALAND

## Abstract

**Background:**

Multidisciplinary collaboration has generally been shown to have positive effects on healthcare but can be difficult to facilitate. This study assessed the effects of a multidisciplinary fibroid clinic on practice patterns and clinician perceptions to better understand drivers of interspecialty collaboration.

**Materials and methods:**

Annual rates of hysterectomies, myomectomies, and uterine fibroid embolizations (UFEs) performed in an urban healthcare system were collected from 2012–2019. Rates of each procedure were compared over time before and after launching a multidisciplinary fibroid clinic at the academic medical center. Referral rates were also compared. The gynecologists and interventional radiologists (IRs) involved in the clinic were interviewed 2 years prior to and after the clinic launch about their approaches to fibroids and perceptions of others who treat this condition. A phenomenological approach was used to identify and compare themes within the interviews by two researchers with excellent inter-rater agreement (κ = 0.80).

**Results and discussion:**

Annual rates of fibroid procedures increased over time (p<0.01) but the relative number of UFEs decreased (p = 0.01). UFE referrals by the clinic gynecologists significantly increased as did the number of combined fibroid procedures (p<0.01). However, the rates of one fibroid procedure relative to others were not different between the clinic and rest of the healthcare system (p = 0.55). Specialty-specific perceptions of fibroid treatments and inter-specialty dynamics did not change. Despite this, clinicians unanimously perceived the clinic and post-clinic practice patterns as positive and distinct from their previous work and relationships between gynecology and IR elsewhere. Limitations of this study included its single clinic design and potential confounder of differences in advertising pre- versus post-clinic.

**Conclusion:**

Creating the right practice environment may be more important for fostering inter-specialty collaboration and work satisfaction than shared mental models or procedural volumes in certain practice settings.

## Introduction

Symptomatic uterine fibroids is a common condition with multiple treatment options primarily offered by two specialties: gynecology and interventional radiology (IR). Historically, fibroids have been the most common benign indication for hysterectomy [1], but both specialties developed uterine-sparing alternatives [2, 3], prompting substantial research and debate comparing these treatment options. These debates were recently fanned by the results of the Fibroids with Either Embolisation or Myomectomy to Measure the Effect on Quality of Life (FEMME) trial, which concluded that myomectomy resulted in superior improvement in quality of life and symptom scores compared to uterine fibroid embolization (UFE) [4]. This has since been criticized by the IR community as problematic, arguing that UFE is equally effective, underutilized, and underdiscussed by gynecologists in counseling their patients [5, 6].

When two or more specialties offer treatments for the same condition, there can be a complex interplay between collaboration and competition [7, 8]. Multidisciplinary collaboration has generally been shown to improve patient outcomes while being cost-effective [9, 10]. Likewise, multidisciplinary fibroid clinics have been shown to deliver high patient satisfaction and increase use of less invasive treatment options [11, 12]. However, the effects of multidisciplinary collaboration on practice patterns and clinician perceptions are less clear. Effective multidisciplinary teams can be difficult to facilitate [13] and challenged by inter-specialty tension [14, 15]. This tension can be distressing and important to consider given the high rates of physician burnout in gynecology and IR [16, 17]. To explore these dynamics and ideal means of facilitating collaboration, this study assessed fibroid practice patterns as well as clinician perceptions before and after the creation of a multidisciplinary fibroid clinic. The authors hypothesized that the clinic would increase inter-specialty referrals and cause the clinicians to have more shared perceptions of fibroid treatments, i.e., shared mental models.

## Materials & methods

The study protocol was reviewed and approved by the Northwestern University Biomedical Institutional Review Board (STU00212421).

### Study design, population, and settings

This study involved an urban healthcare system with an academic medical center (894 beds) as well as community affiliate hospitals. The academic center leadership created a multidisciplinary fibroid treatment clinic staffed by minimally invasive gynecologic surgeons (MIGSs) and interventional radiologists (IRs), which was launched at the beginning of July 2017. To ensure consistent messaging, leadership asked the IR group to stop direct to consumer marketing for UFE, which they had been doing prior to 2017. Other gynecologists and IRs within the healthcare system were not affected by this change; however, the two IRs involved with the multidisciplinary clinic perform most of the UFEs within the system. In order to assess the effects of the clinic on fibroid practice patterns and clinician perceptions, data was collected for at least two years prior to and two years after launching the clinic with a year in between for adjustment. Notably, full-time equivalent counts did not change for the clinicians involved in the clinic during the period studied. The study protocol was reviewed and approved by an institutional review board. Data was reported in accordance with STROBE and SRQR reporting guidelines [18, 19].

### Practice pattern data collection & analysis

Practice pattern data was collected by an institutional data analyst. Collected data included the number of uterine fibroid embolizations, myomectomies, or hysterectomies performed each year for uterine fibroids both within the healthcare system as a whole (Jan. 2012 –Dec. 2019) and the multidisciplinary fibroid clinic (Jul. 2017 –Dec. 2019) based on International Classification of Diseases (ICD) and Current Procedural Terminology (CPT) codes. Primary insurance at the time of the procedure was also collected and categorized as either Medicare/Medicaid or Private/Self-pay. Referrals between the MIGSs and IRs involved in the clinic were also assessed from Jan. 2015 –Dec. 2019.

Practice pattern data was analyzed using Stata 16.1 (StataCorp, LLC, College Station, TX). Procedure and insurance trends over time were assessed via Pearson correlation coefficients. Chi-squared tests were used to compare numbers of procedures pre- and post-clinic as well as between the multidisciplinary clinic and the rest of the healthcare system. An α ≤ 0.05 was considered statistically significant.

### Clinician perceptions data collection & analysis

The two MIGSs and two IRs involved in the multidisciplinary clinic were interviewed about their approach to symptomatic uterine fibroids and perceptions of others who treat this condition two years prior to and after the clinic launch. Interviewees consisted of 3 males and 1 female in practice for 15–38 years at the time of the first interview. Participants were informed about the overall focus of the study at the beginning of the interview and verbal consent was obtained with waiver of documentation to reduce participants’ risk of loss of confidentiality. Interviews were conducted in-person or over the phone, pending participant preference, using a semi-structured approach to establish rapport and facilitate a conversational tone while discussing the same topics across interviews [20]. Clinicians were first asked about their practice and approach to fibroids to allow time for rapport building prior to discussing potentially more taboo topics about other clinicians or specialties that treat fibroids. All interviews were performed by the same qualitative researcher for consistency. The interview script is provided in S1 Table.

Interviews were transcribed verbatim and analyzed using NVivo 12 software (QSR International Inc, Burlington, MA). Initially, inductive content analysis was used to identify themes in descriptions of fibroid treatments and perceptions of the two specialties. Phenomenology and content analysis are well-validated and accepted means of assessing the experiences conveyed in and content of interviews [21, 22]. These methods are more common in the social sciences but have also been used in numerous medical publications [e.g. [23, 24]] and were selected to best capture the complexity of subjects being discussed in the present study. This was performed by two authors and initially yielded 34 unique codes grouped into 4 distinct categories. These researchers then discussed the emerging themes on multiple occasions to construct a central theory that observed differences pre- and post-clinic were best explained by principles of behavioral economics [25, 26] and a social identity approach [15]. Transcripts were then re-coded independently for these principles by the two authors with high coding density and excellent inter-rater agreement (κ = 0.80).

## Results

### Healthcare system practice patterns

**Fig 1:**
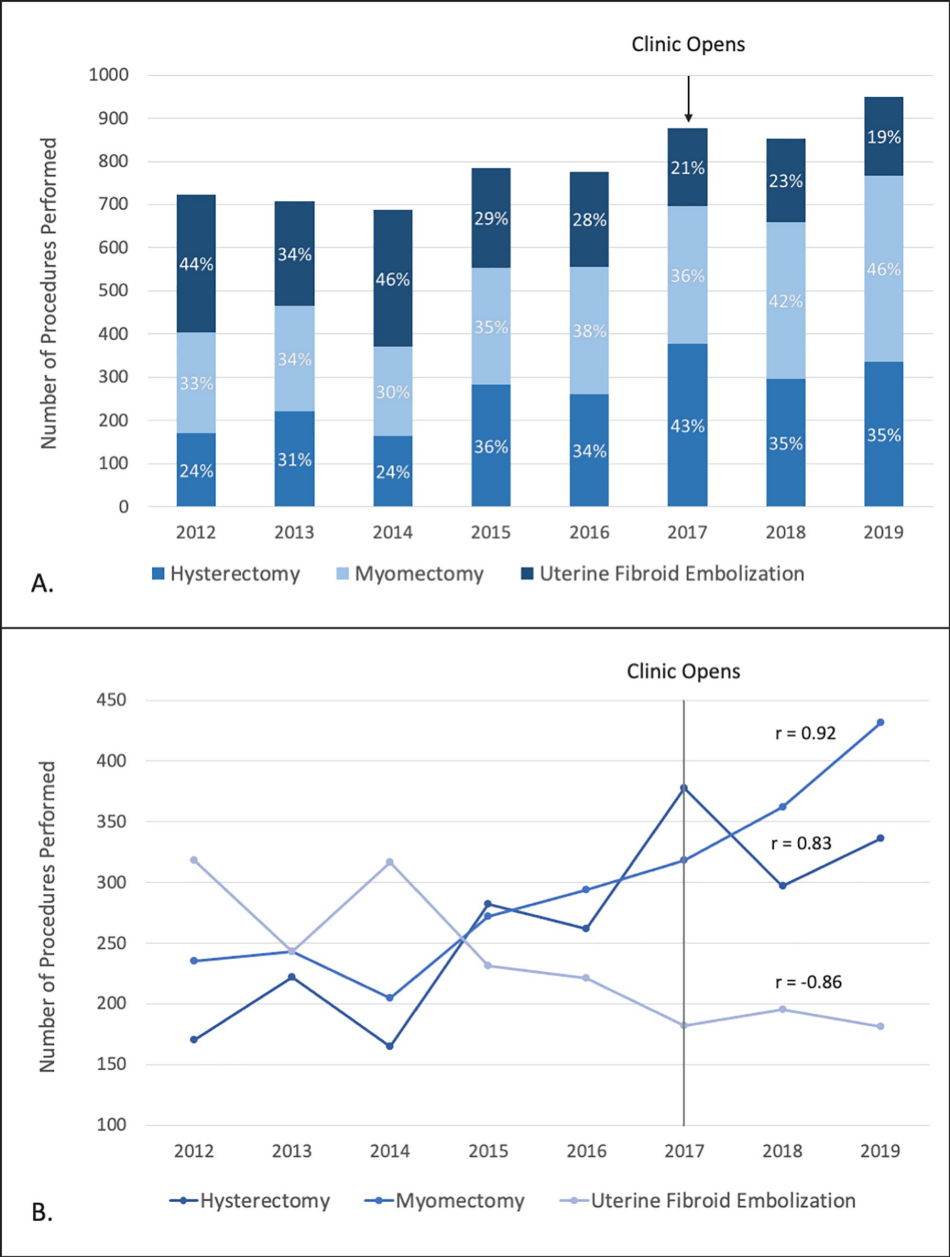
Annual rate of procedures for symptomatic uterine fibroids within the healthcare system as a whole displayed in terms of relative percentages of one procedure compared to others (A) and changes over time with Pearson correlation coefficiencts (B). All coefficients were statistically significant (p ≤ 0.01).

A total of 6361 procedures were performed for symptomatic uterine fibroids from 2012–2019 for 5747 patients (average age 49 ± 9 years). During this period, the number of fibroid procedures increased (r = 0.91, p < 0.01), with a total of 723 procedures performed in 2012 compared to 949 in 2019. The relative percentage of these procedures that were myomectomies and hysterectomies increased (r = 0.92, p < 0.01 and r = 0.83, p = 0.01, respectively), whereas UFEs decreased (r = -0.86, p = 0.01). For example, UFE comprised 44% of procedures for symptomatic uterine fibroids in 2012 compared to 19% in 2019. These trends are summarized in Table 1 and Fig 1.

**Table 1:** Procedure counts in the healthcare system as a whole.

	2012	2013	2014	2015	2016	2017	2018	2019
Open Hysterectomy	30	38	23	100	92	121	81	86
Less invasive † Hysterectomy	140	184	142	182	170	257	216	250
Open Myomectomy	36	26	20	52	71	72	91	98
Less invasive † Myomectomy	199	217	185	220	223	246	271	334
Uterine Fibroid Embolization	318	243	317	231	221	182	195	181
Combined ‡	9	6	7	9	20	22	37	48

* Multidisciplinary clinic launched.

† Less-invasive = laparoscopic or hysteroscopic.

‡ Multiple procedures in the same episode of care, e.g., uterine fibroid embolization followed by myomectomy.

The percentage of procedures paid by Medicare or Medicaid as opposed to private insurance or self-pay did not significantly change for UFEs (r = -0.12, p = 0.74) but increased for myomectomies and hysterectomies (r = 0.94, p < 0.01 and r = 0.74, p = 0.04, respectively). Overall, these percentages tended to be low (≤ 25%) with the majority of fibroid procedures paid via private insurance or self-pay.

### Fibroid clinic practice patterns

**Fig 2:**
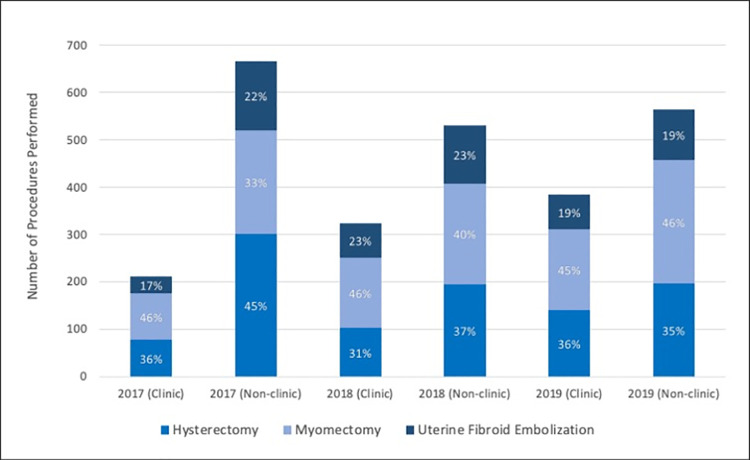
Annual rates of procedures for symptomatic uterine fibroids from the multidiciplinary clinic verses the rest of the healthcare system displayed in terms of relative percentages of one procedure compared to others. There was no significant difference between clinic and non-clinic rates (p = 0.55).

The multidisciplinary clinic saw a total of 2430 patients from Jul. 2017—Dec. 2019 (average age 45 ± 11 years) for symptomatic uterine fibroids and performed 920 procedures in 818 of these patients. The clinic had a steady growth in procedures during this time, increasingly contributing to the overall growth in fibroid procedures observed for the healthcare system as a whole, accounting for 24% (212/878) of all fibroid procedures in 2017 compared to 40% (384/949) in 2019 (p < 0.01). There were also significant increases in the number of UFEs referred by the MIGS involved in the clinic [7% (33/452) in 2015–2016 vs. 30% (114/376) in 2018–2019, p < 0.01] as well as the number of combined fibroid procedures, e.g., UFE followed by myomectomy in the same episode of care (p < 0.01). Nevertheless, the rates of one fibroid procedure relative to others were not different between the clinic and rest of the healthcare system (p = 0.55). These rates are summarized in Fig 2.

### Clinician perceptions

The clinic was universally perceived as positively distinct from pre-clinic practice dynamics and relationships between gynecology and IR elsewhere. Both specialties attributed this to having more of a shared approach, that was easier to facilitate with a shared space and better for patient care. Examples offered included: more informal consults, using pre-procedure magnetic resonance imaging on all patients to guide treatment planning, increased IR involvement in other care such as pelvic nerve blocks, and combined same-day UFE and myomectomy procedures to minimize blood loss and allow for minimally invasive approaches to more complex myomectomy.

Relative to pre-clinic interviews, perceptions of the clinicians in the clinic were more positive; however, perceptions of the other specialty in general did not change. Similarly, specialty-specific perceptions and language surrounding fibroid treatments persisted post-clinic launch, i.e., clinicians perceived more of a shared mental model despite general perceptions of fibroid treatments and those who offer them not changing. Interview coding yielded greater evidence of principles of behavioral economics and social identity theory driving these observations, primarily priming/exposure effect, norms and status quo bias, and ingroup favoritism and fundamental attribution error. Example quotes are provided in Table 2.

**Table 2:** Example quotes.

Perceived shared mental model	You can really build trust, minimize external variables that don’t center on patient outcomes, then I think the clinic just adds to that trust and to that connectivity.–IR 2
	There’s definitely a better understanding of what is the goal. I think it goes both ways, but I get the feeling that they understand more about what we do and what our limitations are as well.–MIGS 1
Priming & Exposure effect	Because we have such a close relationship with our interventional radiologists, I think we probably talk about this way more than the average person… other fibroid specialists are actually somewhat adversarial with interventional radiology… at national meetings, they’re like, "What? You work with IR?"—MIGS 2
	We approached it [with] new office space from the beginning. We didn’t join them in an existing office space. And I think that perhaps gave us a new sort of appreciation or perspective. You know, these are subtle things. It’s funny, human beings and how they interact, it’s small things.–IR 1
Norms & Status quo bias	There probably is a tendency for most IRs to lean towards UFE for patients that reach their doorway and just for the MIG surgeons or for our gynecologists, most probably favor myomectomy–IR 2
In-group favoritism & Fundamental attribution error	I think from a counseling perspective, very few of us counsel appropriately and fairly, and I’m not saying I do it perfectly by any means, but I do feel like I’m a lot more conscientious about counseling and presenting options and presenting them fairly than probably the average OB/Gyn–MIGS 1
	[Re lack of UFE referrals from gynecology] It’s either purely economic, which I suspect is largely the issue or …they live in such an insular world that they literally can’t think outside that world.–IR 1

## Discussion

The results of this mixed methods study suggest that creating a multidisciplinary fibroid clinic positively affected MIGSs’ and IRs’ perceptions of their work and inter-specialty dynamics despite not changing their general perceptions of fibroid treatments and a relative decrease in the number of IR procedures. UFE referrals from the MIGS involved in the clinic increased as did the number of combined MIGS-IR interventions, so the observed decrease in UFEs may have been driven by the IRs stopping direct to patient advertising for UFE. Nevertheless, the IRs involved in clinic perceived it as positively different from previous practice dynamics and relationships between gynecology and IR elsewhere. These results highlight the importance of practice environments for increasing collaboration and work satisfaction, perhaps more than creating shared mental models or procedural volumes in certain practice settings.

Previous work has illustrated a complex interplay between conscious and subconscious aspects of work satisfaction and practice patterns. Medical professionals are often driven by more than reimbursement, particularly a sense of autonomy, mastery, and purpose in their work that can be undermined or fostered by one’s environment [27, 28]. This sense of mastery and purpose is closely tied to one’s professional identity, which varies across specialties just like other professional groups [15, 29]. When inter-specialty dynamics threaten one’s sense of professional identity, it can lead to tension, dissatisfaction, and conflict; however, these social factors of satisfaction often occur subconsciously [14, 30].

Likewise, neuroscientists have described two general systems of decision making: fast automatic processes based on assumptions and heuristics and slow reflective processes that are more conscious and rational [31]. The former decompresses cognitive load, like a cognitive reflex, but is more vulnerable to bias and errors [32]. For example, a clinician may order prophylactic cefazolin before a procedure without much thought because that is what they always do before that procedure, but a new clinical scenario would prompt greater conscious deliberation merely because it is unfamiliar. Often interventions to change behavior in healthcare target reflective rather than automatic processes, such as a campaign to educate a specialty about another’s procedure to increase its use. However, there has been growing recognition of the importance of autonomic processes [25, 33]. Examples highlighted in the results of the present study (Table 2) include a tendency to continue practicing as one has previously and act in accordance with the majority of one’s social group or specialty (“norms” and “status quo bias”). An example of this would be a gynecologist tending recommend myomectomy over UFE for symptomatic uterine fibroids because that is what they have done previously and is common practice among their colleagues and mentors. There is also a human tendency to view one’s own social group more positively and attribute the actions of others to their character or personality (“ingroup favoritism” and “fundamental attribution error”) [15, 25, 26]. An example of this would be IRs favoring the IR approach to fibroids and assuming that differing practices are driven by greed.

The reflective processes of fibroid treatment decision making did not change in the current study, yet UFE referrals and combined procedures increased. This was likely driven by creation of a new, neutral environment (the clinic), which decreased previous friction inhibiting further collaboration, i.e., the clinic made collaboration sufficiently easy to overcome competing subconscious processes such as norms or in-group favoritism [15, 25]. Despite the relative volume of UFE decreasing, the perceived quality of work and dynamics increased, likely contributing to a sense of mastery and purpose and increasing perceived work satisfaction. Additionally, the IRs described the clinic facilitating involvement in other areas of gynecologic care, such as pelvic nerve blocks, so rather than solely competing for the same cases, a shared space and collaborative approach allowed for growth and volume for both IR and MIGS.

On another level, the clinic likely fostered a new positive shared identity (i.e., members of the fibroid clinic) separate from MIGSs’ and IRs’ distinct professional identities, which has previously been described as an important part of interdisciplinary collaboration [34]. Since these distinct identities are not readily differentiated consciously, the clinicians perceived this as having more of a shared mental model despite their descriptions of their approaches to fibroids not substantially changing pre- versus post-clinic. Admittedly, the clinic likely did facilitate more of a shared understanding of fibroid treatments as described by the interviewees, but the effects of the practice environment was more prevalent in the interview coding.

This study had important limitations including its single system design, limiting its external validity. The clinic was created at the academic center in the system where clinicians’ salaries are less tied to volumes. This was also likely key in facilitating collaboration and may undermine similar efforts in other practice settings. Additionally, the number of IRs and MIGSs involved in the clinic were small and thus may not be representative of other IRs and MIGSs. However, the specialty-specific perceptions described are reflected in previous work and descriptions in opinion pieces by the two specialties [6, 14, 35]. Practice patterns can also be affected by a myriad of variables, all of which may not have been captured in this study. The study tried to adjust for this complexity by comparing objective procedure rates with subjective perceptions of all clinicians involved. Qualitative analyses such as those used in this study are vulnerable to bias from researchers acting as the instruments of data collection and interpretation. This was mitigated by using a single experienced interviewer and comparing the agreement in data interpretation between two independent researchers. Finally, the clinical impact and cost-effectiveness of a multidisciplinary fibroid clinic was not assessed in this study. The clinic may have improved perceived dynamics without improving care or even increasing costs by increasing combined procedures. This is an important consideration for future investigations.

In conclusion, creating a multidisciplinary fibroid clinic staffed by MIGSs and IRs increased fibroid procedural volumes and improved perceived inter-specialty dynamics and work satisfaction despite not changing specialty-specific perceptions of fibroid treatments and the relative number of UFEs decreasing. This highlights the importance of practice environments in shaping inter-specialty dynamics, clinical behavior, and perceived work satisfaction.

## Supporting information

S1 Table Interview script. (DOCX)

## References

[1] Wu JM, Wechter ME, Geller EJ, Nguyen TV, Visco AG. Hysterectomy rates in the United States, 2003. Obstet Gynecol 2007 Nov;110(5):1091–5. doi: 10.1097/01.AOG.0000285997.38553.4b 17978124

[2] Bhave Chittawar P, Franik S, Pouwer AW, Farquhar C. Minimally invasive surgical techniques versus open myomectomy for uterine fibroids. Cochrane Database Syst Rev 2014 Oct 21(10):CD004638. doi: 10.1002/14651858.CD004638.pub3 25331441 PMC10961732

[3] Gupta JK, Sinha A, Lumsden MA, Hickey M. Uterine artery embolization for symptomatic uterine fibroids. Cochrane Database Syst Rev 2014 Dec 26(12):CD005073. doi: 10.1002/14651858.CD005073.pub4 25541260 PMC11285296

[4] Manyonda I, Belli AM, Lumsden MA, Moss J, McKinnon W, Middleton LJ, et al. Uterine-Artery Embolization or Myomectomy for Uterine Fibroids. N Engl J Med 2020 Jul 30;383(5):440–51. doi: 10.1056/NEJMoa1914735 32726530

[5] Kennedy SA, Kachura JR, Mafeld S. The FEMME Trial: At Risk for Misinterpretation and "Fake News". Cardiovasc Intervent Radiol Epub 2021 Jan 5. doi: 10.1007/s00270-020-02755-4 33399926

[6] Makris GC, Butt S, Sabharwal T. Unnecessary hysterectomies and our role as interventional radiology community. CVIR Endovasc 2020 Jul 14;3(1):46. doi: 10.1186/s42155-020-00138-x 32666223 PMC7359980

[7] Serra H. Medical technocracies in liver transplantation: drawing boundaries in medical practices. Health (London) 2010 Mar;14(2):162–77. doi: 10.1177/1363459309353297 20164164

[8] Rodwin MA. Medicine, money, and morals: physicians’ conflicts of interest. New York: Oxford University Press; 1993.

[9] Siaw MYL, Malone DC, Ko Y, Lee JY. Cost-effectiveness of multidisciplinary collaborative care versus usual care in the management of high-risk patients with diabetes in Singapore: Short-term results from a randomized controlled trial. J Clin Pharm Ther 2018 Dec;43(6):775–83. doi: 10.1111/jcpt.12700 29696669

[10] Davis MJ, Luu BC, Raj S, Abu-Ghname A, Buchanan EP. Multidisciplinary care in surgery: Are team-based interventions cost-effective? Surgeon 2020 Mar 24. 10.1016/j.surge.2020.02.005 32220537

[11] Tan N, McClure TD, Tarnay C, Johnson MT, Lu DS, Raman SS. Women seeking second opinion for symptomatic uterine leiomyoma: role of comprehensive fibroid center. J Ther Ultrasound 2014;2:3. doi: 10.1186/2050-5736-2-3 25512867 PMC4265989

[12] Josephson RJ, Hatfield J, Forbes MM. A Preliminary Analysis of a Multidisciplinary Fibroid Clinic Patient Satisfaction and Demographics [10F]. Obstetrics & Gynecology 2020;135:63S.

[13] Edmondson AC, Harvey J-F. Cross-boundary teaming for innovation: Integrating research on teams and knowledge in organizations. Human Resource Management Review 2018 2018/12/01/;28(4):347–60.

[14] Keller EJ, Crowley-Matoka M, Collins JD, Chrisman HB, Milad MP, Vogelzang RL. Specialty-Specific Values Affecting the Management of Symptomatic Uterine Fibroids. J Vasc Interv Radiol 2017 Mar;28(3):420–8. doi: 10.1016/j.jvir.2016.11.008 28082073

[15] Kreindler SA, Dowd DA, Dana Star N, Gottschalk T. Silos and social identity: the social identity approach as a framework for understanding and overcoming divisions in health care. Milbank Q 2012 Jun;90(2):347–74. doi: 10.1111/j.1468-0009.2012.00666.x 22709391 PMC3460209

[16] Kravitz RL, Leigh JP, Samuels SJ, Schembri M, Gilbert WM. Tracking career satisfaction and perceptions of quality among US obstetricians and gynecologists. Obstet Gynecol 2003 Sep;102(3):463–70. doi: 10.1016/s0029-7844(03)00666-5 12962925

[17] Bundy JJ, Hage AN, Srinivasa RN, Gemmete JJ, Lee E, Gross JS, et al. Burnout among interventional radiologists. Journal of Vascular and Interventional Radiology 2020;31(4):607–13. e1. doi: 10.1016/j.jvir.2019.06.002 31345730

[18] von Elm E, Altman DG, Egger M, Pocock SJ, Gotzsche PC, Vandenbroucke JP, et al. The Strengthening the Reporting of Observational Studies in Epidemiology (STROBE) statement: guidelines for reporting observational studies. Ann Intern Med 2007 Oct 16;147(8):573–7. doi: 10.7326/0003-4819-147-8-200710160-00010 17938396

[19] O’Brien BC, Harris IB, Beckman TJ, Reed DA, Cook DA. Standards for reporting qualitative research: a synthesis of recommendations. Academic medicine: journal of the Association of American Medical Colleges 2014 Sep;89(9):1245–51. doi: 10.1097/ACM.0000000000000388 24979285

[20] Spradley JP. The ethnographic interview. New York: Holt, Rinehart and Winston; 1979.

[21] Corbin JM, Strauss AL. Basics of qualitative research: techniques and procedures for developing grounded theory. Fourth edition. ed. Los Angeles: SAGE; 2015.

[22] Creswell JW, Poth CN. Qualitative inquiry & research design: choosing among five approaches. Fourth edition. ed. Los Angeles: SAGE; 2018.

[23] Cook DA, Holmboe ES, Sorensen KJ, Berger RA, Wilkinson JM. Getting maintenance of certification to work: a grounded theory study of physicians’ perceptions. JAMA Intern Med 2015 Jan;175(1):35–42. doi: 10.1001/jamainternmed.2014.5437 25365596

[24] Kline CC, Park SE, Godolphin WJ, Towle A. Professional Identity Formation: A Role for Patients as Mentors. Academic medicine: journal of the Association of American Medical Colleges 2020 Oct;95(10):1578–86. 32618605 10.1097/ACM.0000000000003561

[25] King D, Greaves F, Vlaev I, Darzi A. Approaches based on behavioral economics could help nudge patients and providers toward lower health spending growth. Health Aff (Millwood) 2013 Apr;32(4):661–8. doi: 10.1377/hlthaff.2012.1348 23569045

[26] Emanuel EJ, Ubel PA, Kessler JB, Meyer G, Muller RW, Navathe AS, et al. Using Behavioral Economics to Design Physician Incentives That Deliver High-Value Care. Ann Intern Med 2016 Jan 19;164(2):114–9. doi: 10.7326/M15-1330 26595370

[27] Pink DH. Drive: the surprising truth about what motivates us. New York, NY: Riverhead Books; 2009.

[28] De Brantes F. Physician Payment: Forget Carrots And Sticks, It’s Motivation. Health Affairs 2013 Aug. doi: 10.1377/hblog20130807.033633

[29] Becher T. Academic tribes and territories: intellectual enquiry and the cultures of disciplines. Milton Keynes England; Bristol, PA., USA: Society for Research into Higher Education: Open University Press; 1989.

[30] Petriglieri JL. Under threat: Responses to and the consequences of threats to individuals’ identities. Academy of Management Review 2011;36(4):641–62.

[31] Kahneman D. Thinking, fast and slow. 1st pbk. ed. New York: Farrar, Straus and Giroux; 2013.

[32] Saposnik G, Redelmeier D, Ruff CC, Tobler PN. Cognitive biases associated with medical decisions: a systematic review. BMC Med Inform Decis Mak 2016 Nov 3;16(1):138. doi: 10.1186/s12911-016-0377-1 27809908 PMC5093937

[33] Wang SY, Groene O. The effectiveness of behavioral economics-informed interventions on physician behavioral change: A systematic literature review. PLoS One 2020;15(6):e0234149. doi: 10.1371/journal.pone.0234149 32497082 PMC7272062

[34] Fiol CM, Pratt MG, O’Connor EJ. Managing Intractable Identity Conflicts. Academy of Management Review 2009;34(1):32–55.

[35] Mara M, Kubinova K. Embolization of uterine fibroids from the point of view of the gynecologist: pros and cons. Int J Womens Health 2014;6:623–9. doi: 10.2147/IJWH.S43591 25018653 PMC4074023

