# Supplementary material for: The power of proximity: Effects of a multidisciplinary fibroid clinic on inter-specialty perceptions and practice patterns
Source: PLoS One. 2022 Jan 25;17(1):e0263058. doi: 10.1371/journal.pone.0263058 (PMC8789146; doi:10.1371/journal.pone.0263058)
Supplement: S1 Table — (DOCX) [file pone.0263058.s001.docx]

**S1 Table:** Interview Script

| How about we start with you telling me about your practice?  Can you tell me about your approach to symptomatic uterine fibroids?  Is that similar to other [gynecologists / IRs]?  What’s about other specialties that treat these patients and the procedures they offer? What’s your relationship with them? What do you think of those procedures?  Is that similar to other [gynecologists / IRs]?  [Follow up] What do you think drives that? Why is that? Can you give me an example?  [Post-clinic] What about the new fibroid clinic, has that changed things? How so? |
| --- |
